# Solubility and Pharmacokinetic Profile Improvement of Griseofulvin through Supercritical Carbon Dioxide-Assisted Complexation with HP-*γ*-Cyclodextrin

**DOI:** 10.3390/molecules28217360

**Published:** 2023-10-31

**Authors:** Yili Ding, Wutong Cui, Zhiyuan Zhang, Yanzhi Ma, Charles Ding, Yikai Lin, Zhe Xu

**Affiliations:** 1 College of Science and Technology, Wenzhou-Kean University, Wenzhou 325060, China; 2 Wenzhou Municipal Key Laboratory for Applied Biomedical and Biopharmaceutical Informatics, Wenzhou-Kean University, Wenzhou 325060, China; 3 Zhejiang Bioinformatics International Science and Technology Cooperation Center, Wenzhou-Kean University, Wenzhou 325060, China; 4 Dorothy and George Hennings College of Science, Mathematics and Technology, Kean University, 1000 Morris Ave, Union, NJ 07083, USA; 5 Life Science Department, Foshan University, Foshan 528231, China; 6 Keck School of Medicine of USC, University of Southern California, Los Angeles, CA 90089-1149, USA

**Keywords:** griseofulvin, HP-*γ*-cyclodextrin, inclusion complex, preparation, water solubility, in vitro and in vivo PK study

## Abstract

Since griseofulvin was marketed as a non-polyene antifungal antibiotic drug in 1958, its poor water solubility has been an issue for its wide applications, and over the last sixty years, many attempts have been made to increase its water solubility; however, a significant result has yet to be achieved. Through supercritical carbon dioxide-assisted cyclodextrin complexation with the addition of a trace amount of water-soluble polymer surfactant, the griseofulvin inclusion complex with HP-*γ*-cyclodextrin was prepared and confirmed. The 1:2 ratio of griseofulvin and HP-*γ*-cyclodextrin in the complex was determined based on its NMR study. After complexation with HP-*γ*-cyclodextrin, griseofulvin’s water solubility was increased 477 times compared with that of griseofulvin alone, which is the best result thus far. The complex showed 90% of griseofulvin release in vitro in 10 min, in an in vivo dog pharmacokinetic study; the *C*_max_ was increased from 0.52 µg/mL to 0.72 µg/mL, AUC_0–12_ was increased from 1.55 μg·h/mL to 2.75 μg·h/mL, the clearance was changed from 51.78 L/kg/h to 24.16 L/kg/h, and the half-life time was changed from 0.81 h to 1.56 h, indicating the obtained griseofulvin complex can be a more effective drug than griseofulvin alone.

## 1. Introduction

**Figure 1:**
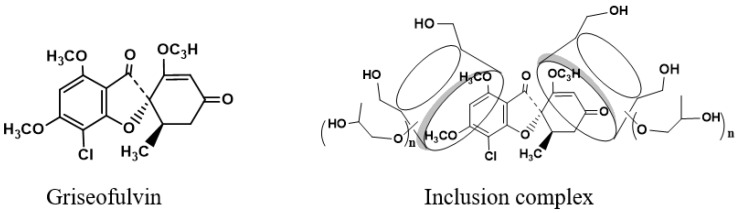
Structure of griseofulvin and its inclusion complex with HP-*γ*-cyclodextrin.

Griseofulvin, structured as (1′S,6′R)- 7-chloro- 2′,4,6-trimethoxy- 6′-methyl- 3H,4′H-spiro [1-benzofuran- 2,1′-cyclohex [2]ene]-3,4′-dione, as shown in Figure 1, is a non-polyene antifungal antibiotic that can strongly inhibit fungal cell mitosis and interfere with fungal DNA synthesis and bind to tubulin to prevent fungal cell division, and it has been widely used in the treatment of fungal infections of the skin and stratum corneum since 1958 [1]. Griseofulvin’s low water solubility (8.64 mg/L) and high permeability group it into class II of the biopharmaceutics classification system [2], and its low dissolution rate in the gastrointestinal tract reduces its bioavailability and clinical efficacy. Griseofulvin solubility in different solvents is affected by the temperature [3]. Therefore, how to improve the solubility and dissolution of griseofulvin has become a key point in clinical practice.

Griseofulvin processed into 3~5 μm particles by using co-precipitation, freeze-drying, or solid dispersion shows water solubility improvement. When its particle size decreases, the solubility will increase [4]; therefore, many methods were developed to transfer griseofulvin powder to nanocrystals in the range of 100–1000 nm [5]. Through milling, griseofulvin nanoparticles with a size of 145 nm were obtained and showed a 97% drug release in just 2 min [6]. Through high-pressure homogenization and spray drying, griseofulvin nanoparticles with a particle size of 60 nm were prepared in the presence of saccharide [7]; the griseofulvin nanoparticles with a size of 16 nm from its acetone and water solution in the presence of stabilizer improved the water solubility by nearly 24 times and achieved almost 100% drug release in 60 min [8]. Through supercritical fluid techniques, griseofulvin submicron particles of 200–500 nm were obtained and showed 80% drug release within 90 min [9]. Through a freeze-drying process, the phospholipid griseofulvin nanoparticles were obtained and showed a seven-fold improvement in drug release in 5 h [10]; griseofulvin nanoparticles of 85 nm from the emulsion solvent diffusion method showed a six-fold increase in water solubility and achieved 85% of drug release within 1 min [11]. Overall, even with great efforts in the preparation of griseofulvin nanoparticles, the water solubility of griseofulvin is still not significantly improved.

Since 1988, cyclodextrins have been used to form the complex with griseofulvin to increase griseofulvin’s water solubility. It was reported that when *α*-cyclodextrin, *β*-cyclodextrin, dimethyl-*β*-cyclodextrin, and *γ*-cyclodextrin were formed in complexes with griseofulvin by using kneading, spray, physical mixing, or precipitation methods, *γ*-cyclodextrin was found to give the best result for an increase in water solubility [12,13,14,15,16]; the solubility, dissolution rate, and bioavailability of griseofulvin in complex were affected by the complexation temperature, surfactants, and the ratio of cyclodextrin and the drug [17,18,19,20]. It was reported that during the formation of the complex, HP-*β*-cyclodextrin could effectively inhibit the particle growth and stabilize the suspensions; in total, 72% of the griseofulvin was precipitated, while the rest of the griseofulvin formed the inclusion complex. The mixture showed improvements in the release rate and water solubility by 9.9 and 11.5 times, respectively [21,22,23]. When the solution of griseofulvin in the mixture of acetone and ethanol was treated with *β*-cyclodextrin, griseofulvin submicron particles from 600 to 900 nm were obtained and showed marked improvement in dissolution velocity [5].

*β*-Cyclodextrins-grafted polymers such as poloxamer-hydroxyethyl cellulose-*α*-cyclodextrin gel [24], polyvinyl alcohol-styrylpyridinium/*β*-cyclodextrin nanofibers [25], and *β*-cyclodextrin-based nano-sponges were complexed with griseofulvin [26], and griseofulvin’s dissolution rate and oral bioavailability were improved; griseofulvin encapsulated by a phospholipid bilayer showed two-times-better bioavailability in rats compared with that of a griseofulvin suspension alone [27]; however, the use of polymers would lead to scale-up and quality control issues, which will limit their applications.

Griseofulvin loaded on silica particles [28] or oxidized mesoporous silicon nanoparticles [29] showed improvements in drug release, but the accumulation of silica nanoparticles in organs presents a challenge [30].

Continuing our research on drug inclusion complexes with cyclodextrin derivatives [31,32,33,34,35], we were further interested in improving the water solubility of griseofulvin by forming a cyclodextrin inclusion complex. In this communication, we would like to report the preparation of the inclusion complex and its in vitro and in vivo pharmacokinetic study in detail.

## 2. Results and Discussion

**Figure 2:**
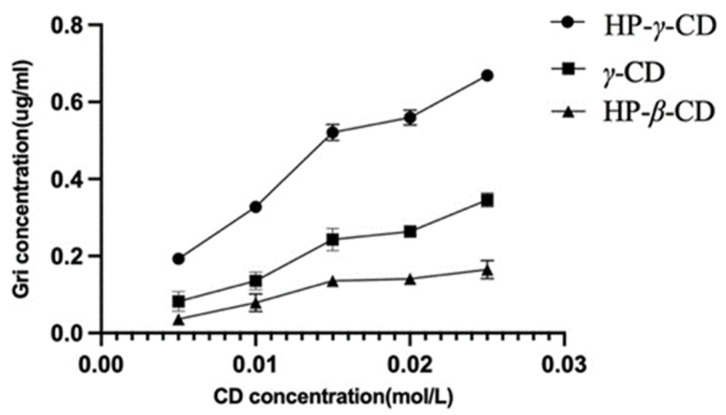
Solubility of griseofulvin in different cyclodextrin solutions.

The UV spectroscopy of the solution of griseofulvin in the mixture of water and ethanol was recorded to obtain griseofulvin’s maximum UV absorption wavelength (292 nm) for its HPLC analysis. Through the phase solubility studies of griseofulvin in the water solutions of HP-*β*-cyclodextrin, *γ*-cyclodextrin, and HP-*γ*-cyclodextrin with different concentrations, it was found that when the concentration of HP-*γ*-cyclodextrin was increased and the solubility of griseofulvin was improved the most. The phase solubility curves were classified as A_L_ types (Figure 2) according to the description from Higuchi and Connors [36].

Complexation between griseofulvin and the cyclodextrin cavity is primarily determined by the tightness of the fit related closely to the size and shape of the cyclodextrin and griseofulvin; *β-* or *γ*-cyclodextrins corresponding to 7 or 8 glucopyranose units have cavity diameters of 6.0–6.5 and 7.5–8.3 A, respectively [37]. Normally, *β*-cyclodextrin can complex with aromatics and heterocycles, and *γ*-cyclodextrin can complex with larger molecules including macrocycles and steroids. If the size of the guest is not perfect, the guest molecule will not fit the cyclodextrin cavity properly, and the interaction between the host–guest and cyclodextrin will decrease [38].

Griseofulvin is approximately 8.972Å in width and 19.884Å in length [39]; consequently, the internal diameter of the *β*-cyclodextrin cavity is too small for the inclusion of the griseofulvin molecule, the internal diameter of the *γ*-cyclodextrin cavity is suitable for accommodation of the griseofulvin molecule; HP-*γ*-cyclodextrin has a better water solubility than *γ*-cyclodextrin and better complexation effects with griseofulvin.

The association constants of the complexation were calculated as *K*_1_ (HP-*β*-cyclodextrin) = 24.7, *K*_2_ (*γ*-cyclodextrin) = 196.1, and *K*_3_ (HP-*γ*-cyclodextrin) = 377.2 from the plot using the following Equation (1), where [S_0_] is the intrinsic solubility of griseofulvin and the slopes of initial straight lines were obtained from phase solubility diagrams.

*K* = slope/S_0_(1 − slope) (1)

Spray drying, grinding, the ultrasonic method, the microwave reaction, using an autoclave reactor, and the water solution method were attempted to prepare the inclusion complex of griseofulvin with HP-*γ*-cyclodextrin, and it was found that the water solution method through supercritical carbon dioxide assistance is a more efficient method than others for comparing the water solubility of griseofulvin.

The inclusion conditions with different molar ratios of griseofulvin and HP-*γ*-cyclodextrin of 1:1, 1:2, or 1:3 at 40 °C, 50 °C, or 60 °C for 10 h, 15 h, or 20 h with stirring speed at 800 r/min, 1000 r/min, or 1200 r/min, respectively, were tried to make the inclusion complex in the mixture of water and acetonitrile with the assistance of supercritical carbon dioxide through statistical orthogonal design strategy. After filtration and lyophilization, the inclusion complex from each condition was obtained for HPLC analysis, and the best complex was obtained from the 1:1 molar ratio of griseofulvin and HP-*γ*-cyclodextrin at 50 °C for 20 h under 1200 r/min stirring speed as a solid with 81% of inclusion yield and 2% of inclusion rate. The water solubility of griseofulvin in the complex was 2.95 mg/mL which is 343-fold higher than that of griseofulvin (0.0086 mg/mL).

Cyclodextrins and cyclodextrin complexes are known to self-associate to form aggregates or micelle-like structures consisting of two to several hundred cyclodextrin molecules and/or cyclodextrin complexes [40], and cyclodextrin aggregates can solubilize lipophilic water-insoluble drugs through non-inclusion complexation [41]. However, the larger cyclodextrin aggregates will limit their water solubility, and water-soluble polymer surfactants can stabilize all kinds of aggregates and micelle-like structures, as well as drug/cyclodextrin complexes [42,43], and anionic and cationic molecules can play similar roles [44]. Griseofulvin may form both inclusion and non-inclusion complexes with HP-*γ*-cyclodextrin as two types of complexes exist in aqueous solutions; after adding a small amount (0.25%) of water-soluble polymers or organic salts such as hydroxypropyl methylcellulose, sodium salicylate, cellulose, sodium carboxymethyl, polyethylene glycol 4000, polyvinylpyrrolidone, hexadimethrine bromide, sodium dodecyl sulfate, tris(hydroxymethyl)aminomethane, sodium acetate, and benzalkonium chloride, the cyclodextrin complexation reaction mixture was worked up and analyzed by HPLC. It was found that the water solubility of griseofulvin in these complexes with a trace amount of water-soluble polymers and organic salts was in the range of 2.50 mg/mL to 4.105 mg/mL, and hydroxypropyl methylcellulose could increase the water solubility of griseofulvin the most to 4.105 mg/mL, which is 477-fold higher than that of griseofulvin, and from a water solubility standpoint, this result is thus far the best one [45]. This sample was used for its structure confirmation and in vitro and in vivo pharmacokinetic study. Only a trace amount of hydroxypropyl methylcellulose (0.25% based on the amount of griseofulvin) existed in the complex, and the FTIR and proton NMR spectra of the complex could not exhibit its characteristic signals.

### 2.1. FTIR Study

**Figure 3:**
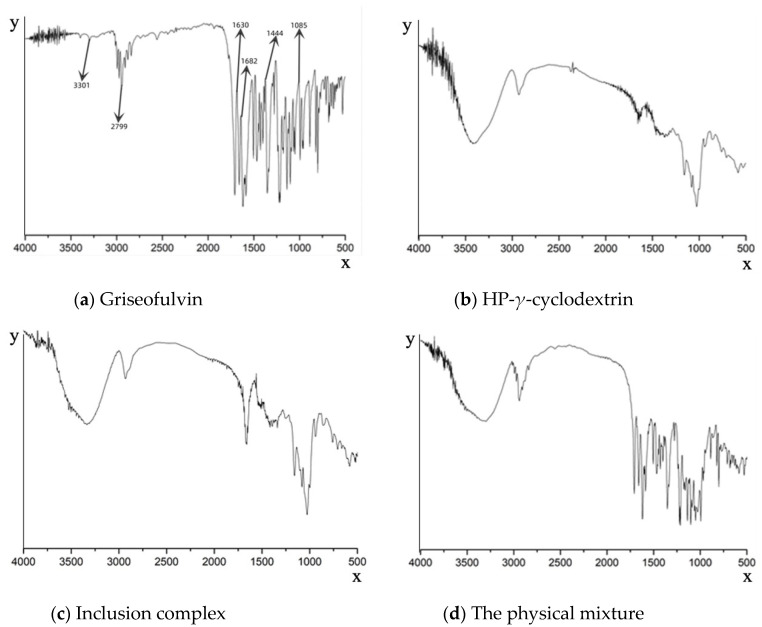
Fourier-transform infrared spectroscopy spectra of griseofulvin (**a**), HP-*γ*-cyclodextrin (**b**), the inclusion complex of griseofulvin with HP-*γ*-cyclodextrin (**c**), and the physical mixture of griseofulvin and HP-*γ*-cyclodextrin (**d**).

The formation of the griseofulvin inclusion complex was confirmed by Fourier-transform infrared spectroscopy as shown in Figure 3. The FTIR spectrum of griseofulvin (Figure 3a) exhibited the significant peaks at 1444.02 cm^−1^ for C-N stretching, a peak at 1682.16 cm^−1^ for C=O vibration, a peak at 1085.35 cm^−1^ for C-O-C stretching, a peak at 3301.77 cm^−1^ for N-H stretching, a peak at 1630.68 cm^−1^ for C=C group vibration, and a peak at 2799.45 cm^−1^ for O-H.

Compared with the inclusion complex, the peak shapes of HP-*γ*-cyclodextrin were unchanged, indicating the structure of HP-*γ*-cyclodextrin in the inclusion complex was unchanged (Figure 3b,c). However, the shapes, positions, and intensity of the peaks corresponding to griseofulvin in the inclusion complex were obviously changed compared with that of griseofulvin, which confirmed the formation of the complex of griseofulvin with HP-*γ*-cyclodextrin, while the infrared spectrum of the physical mixture was just the superposition of the spectra of griseofulvin and HP-*γ*-cyclodextrin (Figure 3d).

### 2.2. NMR Spectral Study

**Figure 4:**
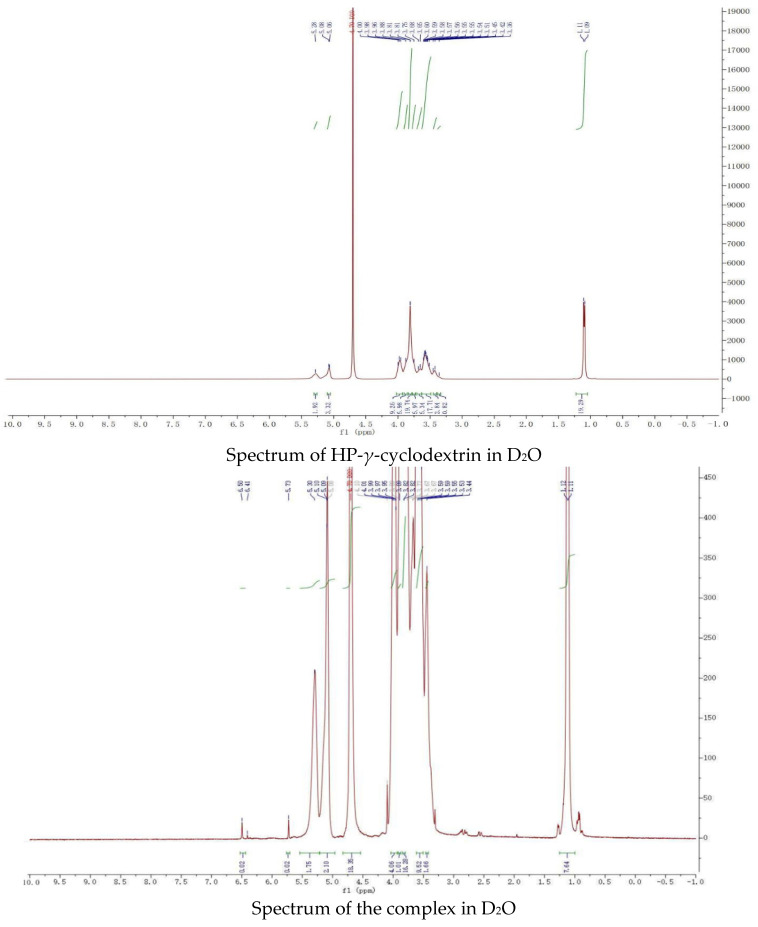

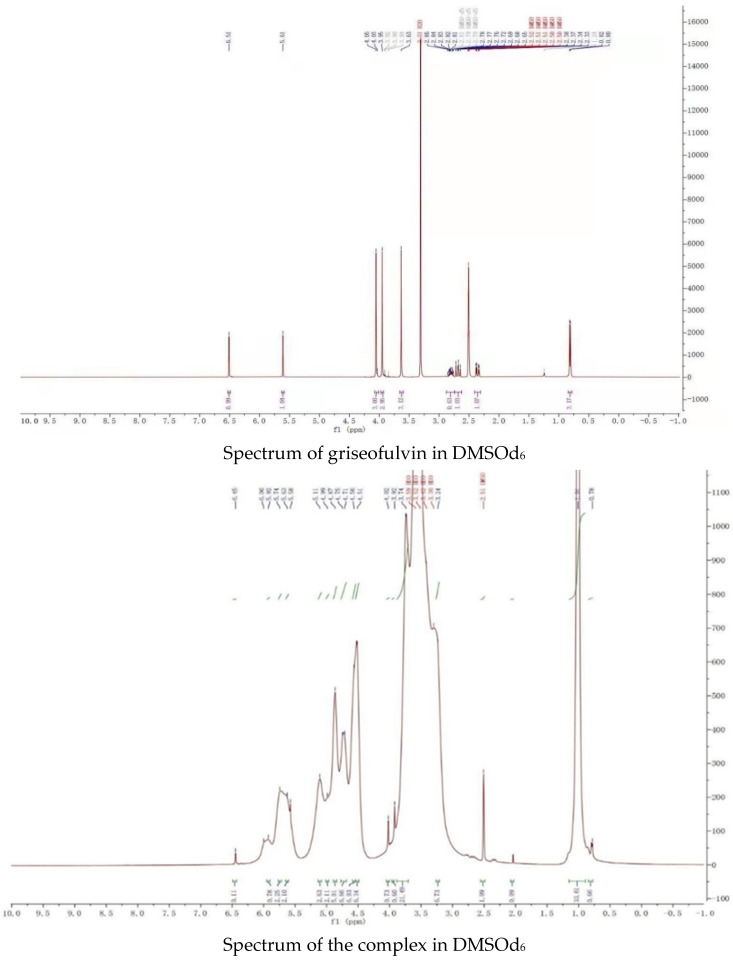
Proton NMR spectra of HP-*γ*-cyclodextrin in D_2_O, griseofulvin in DMSOd_6_, and the inclusion complex in D_2_O and DMSOd_6_.

The proton NMR spectra of griseofulvin (DMSOd_6_), HP-*γ*-cyclodextrin (D_2_O), and the inclusion complex (D_2_O and DMSOd_6_) are shown in Figure 4. The spectrum of the inclusion complex in D_2_O exhibited the signals from griseofulvin at 6.50 (1H, s), 5.73 (1H, s), 2.90–2.78 (2H, m), 2.63 (1H, dd), 1.25 (3H, d, OCH_3_), the signals for three OCH_3_ were overlapped with the signals from HP-*γ*-cyclodextrin in the range of 4.00 ppm to 3.50 ppm. The proton NMR spectrum of the inclusion complex of griseofulvin with cyclodextrin derivative in D_2_O was reported for the first time.

While the proton NMR spectrum of griseofulvin in DMSOd_6_ exhibited signals at 6.51 (1H, s), 5.61 (1H, s), 4.05 (3H, s, OCH_3_), 4.03 (3H, s, OCH_3_), 3.63 (3H, s, OCH_3_), 2.86 (1H, m), 2.79 (1H, dd, *J*_1_ = 12.0 Hz, *J*_2_ = 16.0 Hz), 2.36 (1H, dd, *J*_1_ = 4.0 Hz, *J*_2_ = 16.0 Hz), 0.81 (3H, d, *J* = 8.0 Hz, OCH_3_), the proton NMR data of griseofulvin in the complex in DMSOd_6_ exhibited signals at 6.45 (1H, s), 4.02 (3H, s, OCH_3_), 3.92 (3H, s, OCH_3_), 2.76 (1H, m), 2.65 (1H, dd), 2.33 (1H, dd), 0.785 (3H, d). The chemical shifts of two methoxy groups on the aromatic ring were changed from 4.05 ppm and 4.03 ppm to 4.02 ppm and 3.92 ppm, and the chemical shifts of the aromatic protons were changed from 6.51 ppm to 6.45 ppm, which indicated that the aromatic part of the griseofulvin had the interaction with HP-*γ*-cyclodextrin; the chemical shifts of the methyl group, CH and CH_2_ groups at the cyclohexanone part of griseofulvin were changed from 0.81 ppm, 2.36 ppm, 2.79 ppm, and 2.86 ppm to 0.785 ppm, 2.33 ppm, 2.65 ppm, and 2.76 ppm, which indicated that the cyclohexanone part of griseofulvin had the interactions with HP-*γ*-cyclodextrin. Based on these NMR data, we assume it is impossible for one HP-*γ*-cyclodextrin molecule to have the interactions with the aromatic part and the six-member ring in one griseofulvin molecule; in the complex, it is possible that two HP-*γ*-cyclodextrin molecules are complexed with one griseofulvin molecule as shown in Figure 1.

### 2.3. X-ray Study

**Figure 5:**
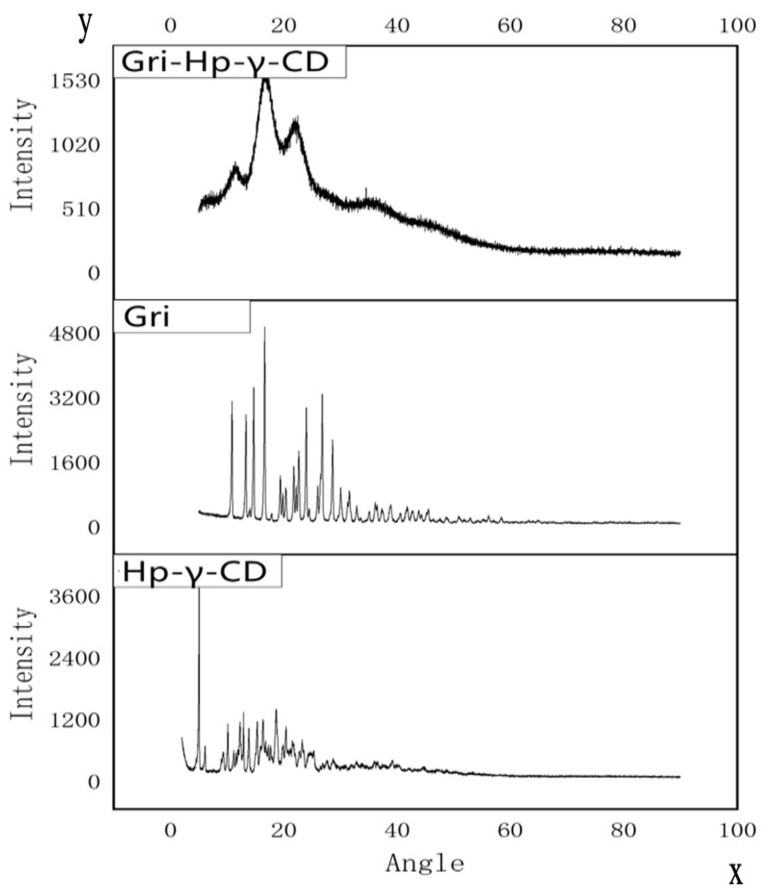
X-ray diffraction patterns of HP-*γ*-cyclodextrin (HP-*γ*-CD), griseofulvin (Gri), and their inclusion complex (Gri-HP-*γ*-CD).

As shown in Figure 5, the X-ray diffraction patterns of griseofulvin and HP-*γ*-cyclodextrin with sharp peaks indicate they are different crystalline compounds; however, the X-ray diffraction pattern of the inclusion complex does not show the characteristic peaks of HP-*γ*-cyclodextrin and griseofulvin completely, which indicates that the product did not contain pure griseofulvin and pure HP-*γ*-cyclodextrin.

### 2.4. SEM Study

**Figure 6:**
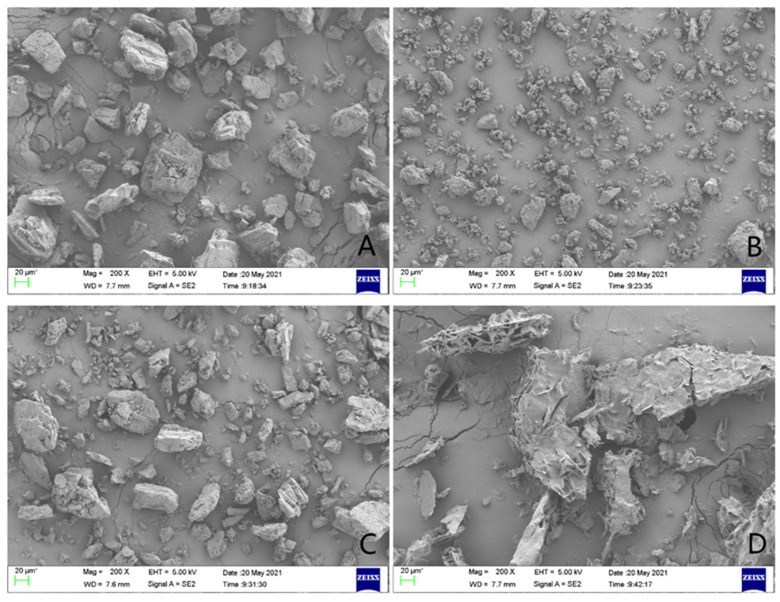
The scanning electron microscope images of HP-*γ*-cyclodextrin (**A**), griseofulvin (**B**), the physical mixture of griseofulvin and HP-*γ*-cyclodextrin (**C**), and the inclusion complex (**D**).

Scanning electron microscopy was used to study the changes in the surface morphology of griseofulvin and its inclusion complex, and their micrographs are shown in Figure 6. When the sample was magnified to 20 μm, the sample of HP-*γ*-cyclodextrin appeared as an uneven block shape (Figure 6A), the sample of griseofulvin appeared as an amorphous shape (Figure 6B), and in their physical mixture, griseofulvin was distributed around the HP-*γ*-cyclodextrin (Figure 6C), while the inclusion complex presented an irregular fragment shape (Figure 6D). The difference between the micrographs of griseofulvin and its complex with HP-*γ*-cyclodextrin confirmed the formation of the inclusion complex directly.

### 2.5. The Dissolution Rate Study

**Figure 7:**
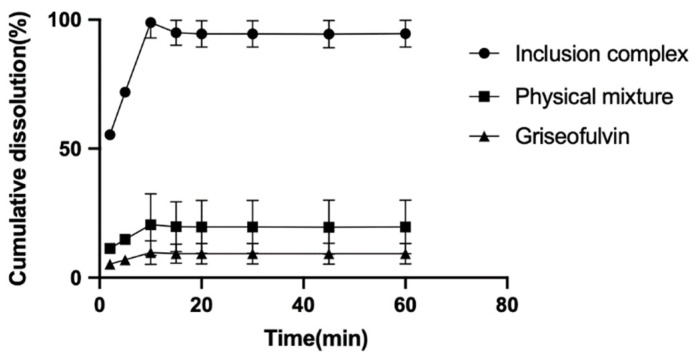
The dissolution rate curves of griseofulvin, the mixture of griseofulvin and HP-*γ*-cyclodextrin, and the inclusion complex.

The dissolution rates of griseofulvin, the physical mixture of griseofulvin and HP-*γ*-cyclodextrin, and their inclusion complex in degassed water with a pH of 7 were examined and are shown in Figure 7. After 10 min, the cumulative dissolution of griseofulvin in water was only near 2%, the cumulative dissolution of the physical mixture of griseofulvin and HP-*γ*-cyclodextrin was about 18%, while the cumulative dissolution of the inclusion complex of griseofulvin with HP-*γ*-cyclodextrin was more than 90%, which was much better than that of griseofulvin alone.

In addition, in the inclusion complexes, griseofulvin is ensconced in the HP-*γ*-cyclodextrin cylinder, the direct contact between griseofulvin and taste sensors is inhibited; therefore, the unpleasant taste of griseofulvin is eliminated.

The in vivo pharmacokinetic study of the complex in dogs was conducted, griseofulvin and its HP-*γ*-cyclodextrin inclusion complex were orally dosed to dogs in two groups (six for each group) at a single dose of 15 mg/kg, the blood samples were collected at 0, 0.25, 0.5, 0.75, 1, 2, 3, 4, 6, 8, 12, 24, 36, 48, and 72 h immediately after drug administration, respectively.

**Figure 8:**
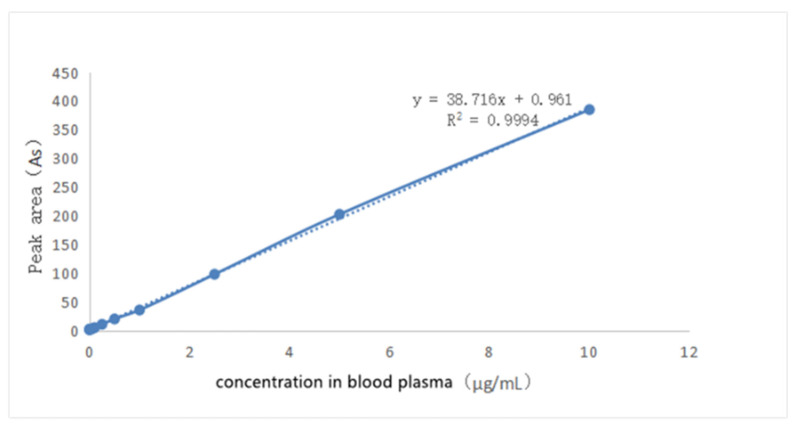
The standard curve of griseofulvin in dog’s blank plasma.

The solution of griseofulvin in acetonitrile (0.1 mg/mL) was added to the blank dog plasma to prepare a series of solutions to obtain the standard working curve shown in Figure 8 through HPLC analysis. When the concentration of griseofulvin in plasma was in the range of 0.05 μg/mL~20 μg/mL, the peak area in HPLC and the concentration of griseofulvin in solution show a linear relationship, and the standard working curve equation was obtained as: Y = 38.716X + 0.961 (R^2^ = 0.9994, Y: peak area, X: concentration). The S/N ≥ 10: LLOD was 0.01 μg/mL, S/N ≥ 3, LLOQ was 0.05 μg/mL.

**Figure 9:**
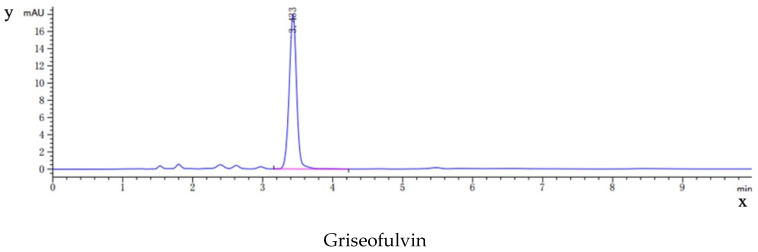

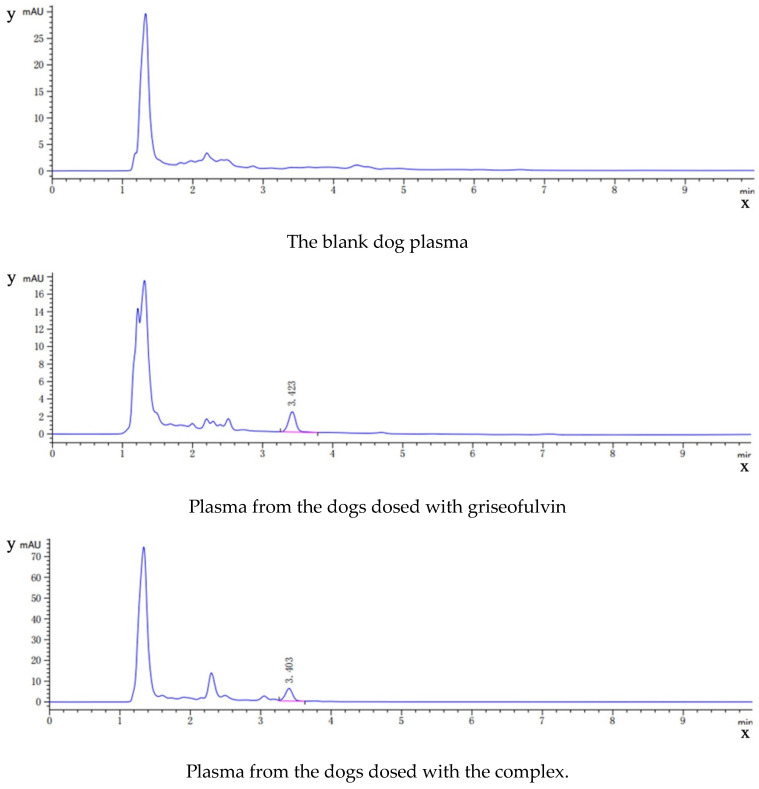
HPLC analysis (concentrations and time) of griseofulvin, blank dog plasma, plasma from the dogs dosed with griseofulvin, and the plasma from the dogs dosed with griseofulvin inclusion complex.

As shown in Figure 9, the HPLC analysis of griseofulvin, the blank dog plasma, the plasma from the dogs dosed with griseofulvin, and the plasma from the dogs dosed with the griseofulvin/HP-*γ*-cyclodextrin inclusion complex indicated that the blank dog plasma did not interfere with the detection of griseofulvin.

The standard solutions of griseofulvin with concentrations of 0.1 μg/mL, 1 μg/mL, and 10 μg/mL in blank plasma were used to determine the recovery rate, intra-assay coefficient of variation, and the inter-assay coefficient of variation, and the recovery rate of griseofulvin in plasma is in the range of 92.32 ± 0.43%~108.75 ± 6.12%, while the intra-assay coefficient of variation is in the range of 0.07~5.63%, and the inter-assay coefficient of variation is in the range of 1.11~3.73%.

**Figure 10:**
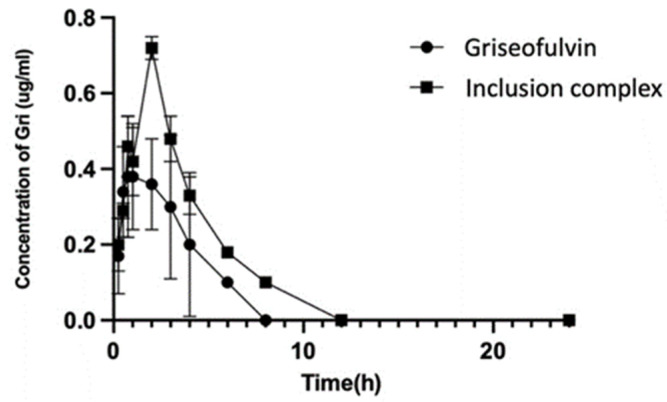
The concentrations of griseofulvin in plasmas from dogs dosed with griseofulvin or its inclusion complex.

The collected plasma samples were used for HPLC analysis, and the results are shown in Figure 10. The HPLC analysis of the samples collected at 24, 36, 48, and 72 h in the drug group and complex group did not show any signals of griseofulvin. DAS 2.0 pharmacokinetic software was used to analyze the experimental data, and the pharmacokinetic parameters (mean ± SD) are listed in Table 1.

After oral administration, the *C*_max_ and *T*_max_ of griseofulvin in the plasma of dogs were found as 0.52 μg/mL at 1.75 h for the griseofulvin group and 0.72 μg/mL at 2.0 h for the griseofulvin inclusion complex group; the AUC_0–12_ in the griseofulvin group was found as 1.55 μg·h/mL and in the inclusion complex group it was found as 2.75 μg·h/mL. Through complexation, the *C*_max_ was increased by 138%, the level of exposure of the griseofulvin was increased by 177%, the delayed *T*_max_ would benefit the distribution of griseofulvin, the clearance of griseofulvin was changed from 51.78 L/kg/h to 24.16 L/kg/h, and the half-life time was changed from 0.81 h to 1.56 h. The increased half-life time and decreased clearance allow griseofulvin to stay longer in the body and have a longer duration of action; these facts indicated that less drug is needed for the same therapeutic effect by using the griseofulvin complex, and the higher water solubility may give a better efficacy [46].

The increased aqueous solubility (477-fold) of the griseofulvin in complexation with HP-*γ*-cyclodextrin, which is the best thus far in griseofulvin’s water solubility study, might be responsible for 90% of the drug release in 10 min, a 177% AUC_0–12_ increase, shorter clearance, and a longer half-life time.

Since 1988, cyclodextrin derivatives have been used to complex with griseofulvin to improve griseofulvin’s water solubility [47]. Thirty-five years later, significant water solubility increasing results have not been found. Through very careful methodology screening, we found the method to prepare the griseofulvin inclusion complex with a 477-fold increase in water solubility. More importantly, we used the proton NMR spectrum of the inclusion complex in D_2_O to confirm the solubility increasing, and based on the NMR data, we are able to know the ratio of griseofulvin and cyclodextrin in the complex for the first time, and the relative bioavailability was increased to 177% through complexation.

## 3. Materials and Methods

### 3.1. Materials and Instruments

Griseofulvin (98%) was obtained from Beijing solebo Technology Co., Ltd. (Beijing, China), HP-*γ*-cyclodextrin (degree of substitution: 4.0–5.6, average WM: 1589) was obtained from Shanghai Yien Chemical Technology Co., Ltd. (Shanghai, China), HP-*β*-cyclodextrin (molecular weight: 1431–1806; degree of substitution: 4.81–4.87) was purchased from Yunan County Yongguang Ring Dextrin Co., Ltd. (Yunan, Guangdong, China); *γ*-cyclodextrin was purchased from Shanghai Yien Chemical Technology Co., Ltd. (Shanghai, China); ^1^H NMR (nuclear magnetic resonance) spectra were obtained on Bruker spectrometer (400 MHz) in D_2_O or DMSOd_6_; the UV (ultraviolet) spectra were obtained in J51903001 ultraviolet visible spectrophotometer instrument from Shanghai Jinghua Technology Instrument Co., Ltd. (Shanghai, China); the FTIR (Fourier-transform infrared spectroscopy) spectra were recorded on a Fourier Transform Infrared Spectrometer IRTracer 100 from Shimadzu, the SEM (scanning electron microscope) images were recorded on a Merlin from German Zeiss company, the X-ray analysis was performed on a D8 Advance X-ray polycrystalline diffractometer from Bruker, the HPLC (high-performance liquid chromatography analysis) was performed on a LC-15C high-performance liquid chromatograph from Shimadzu Enterprise Management (Beijing, China) Co., Ltd. by using an Inertsil ODS-3 C18 (250 mm × 4.6 mm) column, the freeze dryer OLB-FD10S was purchased from OLABO Scientific Co., Ltd. (Guangzhou, Guangdong, China), dogs were gotten from Guangdong Medical Laboratory Animal Center, the blank dog plasma was received from Guangzhou Rui-Te Co., Ltd. (Guangzhou, Guangdong, China) and the low-fat dog foods were obtained from Shanghai Jibai Chong Industrial Co., Ltd. (Shanghai, China).

An ultrasonic reactor, JY92-IIN, was obtained from Ningbo Xinzhi Biotechnology Co., Ltd. (Ningbo, Zhejiang, China), the microcomputer microwave chemical reactor, WBFY-201, was obtained from Gongyi Yuhua Instrument Co., Ltd. (Xinxiang, Henan, China), a high temperature and pressure reaction kettle, BZ-100ML/SC-L, was obtained from Shanghai Baikal Technology Group Co., Ltd. (Shanghai, China), a magnetic stirrer RCT digital was obtained from IKA (Berlin, Germany), the supercritical carbon dioxide reactor BZ-100ML/S0-L was purchased from Baikal Shanghai Intelligent Technology Co., Ltd. (Shanghai, China), and the dissolution tester RC-6 was obtained from Tianjin Xintianguang Analytical Instrument Technology Co. (Tianjin, China).

### 3.2. UV Spectrometry Spectral Study

The UV spectra of the solution of HP-*γ*-cyclodextrin (5 mg) in H_2_O (1 mL) and the solution of griseofulvin (1 mg) in the mixture of water and ethanol (1:1, 1 mL) were recorded in the wavelength range of 200–400 nm by using the mixture of water and ethanol (1:1, 1 mL) as a blank. Griseofulvin had the maximum absorption at 292 nm, while HP-*γ*-cyclodextrin had no absorption at this wavelength; thus, 292 nm was chosen as the wavelength in HPLC analysis of griseofulvin.

### 3.3. HPLC Analysis

A series of griseofulvin solutions from 500 μg/mL to 1 μg/mL diluted from the solution of griseofulvin (5 mg) in acetonitrile (2 mL) were analyzed on HPLC with 20 μL of injection volume to obtain the linear regression equation as Y = 23,582X + 22.861 (coefficient of determination: R^2^ = 0.9999, Y: peak area, X: concentrations from 8 μg/mL to 500 μg/mL). The LLOD was 0.01 μg/mL, and LLOQ was 0.05 μg/mL, the running time for each sample was 10 min, and the concentration of the fresh prepared solution for curve calibration is 0.01 μg/mL.

The HPLC standard curves and sample analysis including plasma samples were performed in the same HPLC instrument with the same C-18 column during a short period of time to avoid systematic errors, and we did not use the internal standard for calibration.

### 3.4. Phase Solubility Curves

The mixtures of griseofulvin (35 mg, 0.10 mmol) in aqueous solutions of HP-*β*-cyclodextrin, *γ*-cyclodextrin, or HP-*γ*-cyclodextrin (0.005 mol, 0.01 mol, 0.015 mol, 0.02 mol, or 0.025 mol in 10 mL of water) were shacked at 25 °C for 48 h in oscillation boxes, respectively, filtered through a 0.22 μm microporous membrane, and analyzed by HPLC to give the phase solubility curves.

### 3.5. Preparation of the Inclusion Complex with Supercritical Carbon Dioxide Assistance

The solution of HP-*γ*-cyclodextrin (1060 mg, 0.6 mmol) in distilled water (8 mL) was added the solution of griseofulvin (105 mg, 0.3 mmol) in acetonitrile (8 mL), stirred at 50 °C, pumped the liquid CO_2_ at the pressure of 8 Mpa, and stirred for 12 h. After releasing the pressure of the reaction slowly, the mixture was evaporated, distilled water added, filtrated, and lyophilized at −58 °C with reduced pressure for 48 h to give the griseofulvin inclusion compound as a solid, which was then stored in a refrigerator at 4 °C under nitrogen. By using the same procedure, a trace amount (0.25%) of one of the short-chain anionic or cationic species was added to the reaction mixture at beginning, and the inclusion complex containing small amount of one of the short-chain anionic or cationic species was obtained.

### 3.6. FTIR Spectral Analysis

The KBr pellets of griseofulvin, HP-*γ*-cyclodextrin, the physical mixture of griseofulvin and HP-*γ*-cyclodextrin, and the inclusion complex were analyzed by FTIR in the range of 500 cm^−1^ and 4000 cm^−1^.

### 3.7. NMR Spectral Study

The proton NMR spectra of the solutions of HP-*γ*-cyclodextrin and the griseofulvin inclusion complex in D_2_O and the solutions of griseofulvin and its inclusion complex in DMSO-d_6_ were recorded at 400 MHz at room temperature.

### 3.8. X-ray Analysis

The X-ray diffraction spectra of griseofulvin, HP-*γ*-cyclodextrin, and their inclusion complex were recorded in conditions of: Cu-k α emission, lynxeye array detector, tube voltage of 40 kV and current of 40 mA, scanning angle range of 3–60 degrees and scanning speed of 0.01/s; each solid sample of griseofulvin, HP-*γ*-cyclodextrin, and their inclusion complex was placed on a magnetic block, sprayed with gold, loaded onto a sample rod, and scanned at accelerating voltage of 5 kV under high vacuum to obtain the surface morphologies with 500-times image magnification and the secondary electronic image type.

### 3.9. SEM Study

The samples of griseofulvin, HP-*γ*-cyclodextrin, their physical mixture, and the inclusion complex were placed on the sample stage, gold-plated, scanned, and pictured under 5 kV of the accelerating voltage, respectively.

### 3.10. The Content of Griseofulvin in the Complex

The inclusion complex (100 mg) was dissolved in deionized water (1 mL) and diluted to the range of 8 μg/mL~500 μg/mL to obtain the content of griseofulvin in complex based on the HPLC analysis data and regression equation.

### 3.11. Water Solubility of Griseofulvin in Complex

The water solubility of griseofulvin in complex was obtained from the HPLC analysis of a saturated aqueous solution of the inclusion complex in water (1 mL).

### 3.12. The Inclusion Rate and Inclusion Yield

The inclusion yield can be obtained from the following formula: inclusion yield (%) = [griseofulvin inclusion complex (mg)/griseofulvin (mg) + HP-*γ*-cyclodextrin (mg)] × 100%.

The inclusion rate can be obtained from the formula: inclusion rate (%) = [griseofulvin in inclusion complex (mg)/griseofulvin (mg)] × 100%.

### 3.13. The Dissolution Rate

The griseofulvin (100 mg), the physical mixture of griseofulvin (100 mg) and HP-*γ*-cyclodextrin (1000 mg), or the complex of griseofulvin with HP-*γ*-cyclodextrin (containing 100 mg of griseofulvin) were used for testing, respectively. From the medium (degassed water: 900 mL) at 37 °C with 100 r/min rotating speed, samples (3 mL for each) at 2, 5, 10, 15, 20, 30, 45, and 60 min were collected for HPLC analysis and followed by the addition of 3 mL of water after each collection, respectively.

### 3.14. In Vivo Pharmacokinetics Study

Healthy dogs (12) around 5 kg were randomly divided into two groups as the griseofulvin group and the complex group, each group containing three male and three female dogs. After fasting for 12 h, water was forbidden for 1 h before dosing. A single oral dose of griseofulvin or its complex was administered to each dog at 15 mg/kg in 10 mL of water. After drug administration, the dog was fasted for food for 4 h and for water for 1 h. The blood samples (2 mL) were collected with disposable EDTA (ethylenediaminetetraacetic acid) anticoagulation syringes at 0, 0.25 h, 0.5 h, 0.75 h, 1 h, 2 h, 3 h, 4 h, 8 h, 12 h, 24 h, 36 h, 48 h, and 72 h, respectively. After being centrifuged at 4000 r/min for 10 min, the plasma samples were stored at −80 °C in a refrigerator.

The plasma sample (0.5 mL) in 1 mL of acetonitrile was vortexed in a 2 mL centrifuge tube for 3 min, centrifuged at 13,000 r/min for 10 min, and the supernatant was collected in a glass tube. The precipitate was extracted with 0.5 mL of acetonitrile two times, and the combined supernatants were centrifuged and dried under nitrogen at 37 °C, 0.5 mL of the mobile phase was added, and the mixture was vortexed for 4 min, filtered, and analyzed by HPLC by using 60:40 of acetonitrile/water with 0.1% acetic acid as the mobile phase with a 1.0 mL/min of flow rate on an Inertsil ODS-3 C18 (250 mm × 4.6 mm) column by UV detector at 290 nm at 30 °C. The recovery rate of griseofulvin in plasma was 92.32 ± 0.43%~108.75 ± 6.12%, the intra-assay coefficient of variation was 1.11~3.73%, and the inter-assay coefficient of variation was 0.07~5.63%.

### 3.15. Standard Curve for Griseofulvin in Dog Plasma

The solutions of griseofulvin in the mobile phase (50 µL) with different concentrations from 0.5, 1, 2.5, 5, 10, 25, 50 to 100 µg/mL were added to 450 µL of the canine blank plasma in a centrifuge tube. After vortexing in a vortexer for 4 min, each sample was analyzed by HPLC, and based on the peak areas and concentrations, the standard curve of griseofulvin in dog plasma was obtained.

### 3.16. Precision

The solutions of griseofulvin in the mobile phase (50 µL) with the concentrations of 2, 50 and 100 µg/mL were added into 450 µL of canine blank plasma, each mixture was vortexed for 3 min, obtaining the griseofulvin solutions in plasma with low, medium, and high concentrations as 0.2, 1, and 5 µg/mL, respectively, three samples were prepared for each concentration, and each sample was analyzed by HPLC three times to obtain the intra-day coefficient of variation. The experiments were repeated for three days to obtain the inter-day coefficient of variance.

### 3.17. Statistical Analysis

Each experiment was carried out in triplicate to obtain the average value, and results are expressed as the mean ± standard deviation. Each point in the pharmacokinetic curve represents the average of six experiments. GraphPad Prism 5.0 software was selected for statistical analysis, including data processing and graphical presentation. Two-tailed t-tests were used to assess the significance of differences in pharmacokinetic parameter values between dogs given pure griseofulvin and dogs given the inclusion complex. An AP value of 0.05 was considered significant for statistical analysis.

## 4. Conclusions

In conclusion, the griseofulvin inclusion complex with HP-*γ*-cyclodextrin was prepared through supercritical carbon dioxide assisted complexation with addition of a tract amount of hydroxypropyl methylcellulose and confirmed by FTIR, NMR, X-ray, and ESM. The ratio of griseofulvin and HP-*γ*-cyclodextrin in the complex was determined as 1:2 based on the proton NMR data. Through the complexation, the water solubility of griseofulvin was increased 477 times compared with that of griseofulvin alone, which is thus far the best result. The complex showed more than 90% of griseofulvin was released in 10 min, its in vivo pharmacokinetic studies showed significant improvement in pharmacokinetic properties comparing with griseofulvin, the *C*_max_ was increased from 0.52 µg/mL to 0.72 µg/mL, AUC_0–12_ was increased from 1.55 μgh/mL to 2.75 μgh/mL, the clearance was changed from 51.78 L/kg/h to 24.16 L/kg/h, and the half-life time was changed from 0.81 h to 1.56 h, which indicated this griseofulvin complex can be used as a potential more effective antifungal, antibiotic, or anti-cancer drug than griseofulvin alone.

## Figures and Tables

**Table 1:** Pharmacokinetic parameters of griseofulvin and its inclusion complex.

Parameters	Unite	Griseofulvin	Complex
*T*_1/2_	h	0.81 ± 0.37	1.56 ± 0.84
*T*_max_	h	1.75 ± 0.98	2.00 ± 0.00
*C*_max_	µg/mL	0.52 ± 0.13	0.72 ± 0.03
AUC_0–12_	h·µg/mL	1.55 ± 0.23	2.75 ± 0.23
CL	L/kg/h	51.78 ± 22.76	24.16 ± 1.90

## Data Availability

Data are available to share.
